# Genomic Comparison and Spatial Distribution of Different *Synechococcus* Phylotypes in the Black Sea

**DOI:** 10.3389/fmicb.2020.01979

**Published:** 2020-08-12

**Authors:** Andrea Di Cesare, Nina Dzhembekova, Pedro J. Cabello-Yeves, Ester M. Eckert, Violeta Slabakova, Nataliya Slabakova, Elisaveta Peneva, Roberto Bertoni, Gianluca Corno, Michaela M. Salcher, Lyudmila Kamburska, Filippo Bertoni, Francisco Rodriguez-Valera, Snejana Moncheva, Cristiana Callieri

**Affiliations:** ^1^National Research Council (CNR), Institute of Water Research (IRSA), Verbania, Italy; ^2^Institute of Oceanology “Fridtjof Nansen” – Bulgarian Academy of Sciences, Varna, Bulgaria; ^3^Evolutionary Genomics Group, Departamento de Producción Vegetal y Microbiología, Universidad Miguel Hernández, San Juan de Alicante, Spain; ^4^Faculty of Physics, Sofia University “St. Kliment Ohridski”, Sofia, Bulgaria; ^5^Biology Centre Czech Academy of Science (CAS), Institute of Hydrobiology, Czechia; ^6^Museum für Naturkunde, Berlin, Germany; ^7^Laboratory for Theoretical and Computer Studies of Biological Macromolecules and Genomes, Moscow Institute of Physics and Technology, Dolgoprudny, Russia

**Keywords:** marine *Synechococcus* spp., *rpoC1*, qPCR, mesopelagic Black Sea, epipelagic Black Sea

## Abstract

Picocyanobacteria of the genus *Synechococcus* are major contributors to global primary production and nutrient cycles due to their oxygenic photoautotrophy, their abundance, and the extensive distribution made possible by their wide-ranging biochemical capabilities. The recent recovery and isolation of strains from the deep euxinic waters of the Black Sea encouraged us to expand our analysis of their adaptability also beyond the photic zone of aquatic environments. To this end, we quantified the total abundance and distribution of *Synechococcus* along the whole vertical profile of the Black Sea by flow cytometry, and analyzed the data obtained in light of key environmental factors. Furthermore, we designed phylotype-specific primers using the genomes of two new epipelagic coastal strains – first described here – and of two previously described mesopelagic strains, analyzed their presence/abundance by qPCR, and tested this parameter also in metagenomes from two stations at different depths. Together, whole genome sequencing, metagenomics and qPCR techniques provide us with a higher resolution of *Synechococcus* dynamics in the Black Sea. Both phylotypes analyzed are abundant and successful in epipelagic coastal waters; but while the newly described epipelagic strains are specifically adapted to this environment, the strains previously isolated in mesopelagic waters are able, in low numbers, to withstand the aphotic and oxygen depleted conditions of deep layers. This heterogeneity allows different *Synechococcus* phylotypes to occupy different niches and underscores the importance of a more detailed characterization of the abundance, distribution, and dynamics of individual populations of these picocyanobacteria.

## Introduction

The Black Sea is a unique marine basin, largely isolated from the global ocean, and characterized by an extensive drainage basin, strong vertical stratification, variable salinity (from 7.3 in epipelagic to ca. 20–22 PSU in mesopelagic waters) and high concentrations of hydrogen sulfide content below 150–200 m (Konovalov et al., 2005; Stanev et al., 2014). These conditions confine aerobic biological activity to the upper 100–150 m of the water column, while the water mass below these depths is usually considered inhospitable and restricted to anaerobic microbes (Zaitsev and Mamaev, 1997; Kucuksezgin and Pazi, 2003). The recent recovery of *Synechococcus* cells living in deep anoxic water layers (Callieri et al., 2019) challenged our knowledge of this part of the Black Sea and its biogeochemical dynamics, and of *Synechococcus*’ role in them.

Picocyanobacteria of the genus *Synechococcus* are small autotrophic unicellular organisms (0.6–2 μm), widespread in aquatic ecosystems (e.g., Callieri et al., 2012; Flombaum et al., 2013; Sohm et al., 2016), possessing light-harvesting phycobilisomes with antenna pigments that enable them to use a wide range of wavelengths and colonize different ecological niches (e.g,. Ahlgren and Rocap, 2006; Six et al., 2007). Despite *Synechococcus’* remarkable adaptability to different salinities (Fuller et al., 2003; Kim et al., 2018), temperatures, Fe and nutrient concentrations (Fuller et al., 2006; Callieri, 2017; Zwirglmaier et al., 2008), the detection of *Synechococcus* in an extreme environment like the deep anoxic Black Sea was unexpected – even at relatively low concentrations. This is because, as oxygenic autotrophs, *Synechococcus* have been usually considered restricted to the photic layer.

Because of their sheer abundance, broad metabolic potential, and widespread distribution, these cyanobacteria are major contributors to global primary production (Flombaum et al., 2013): marine *Synechococcus* alone contributes to around 21% of the total primary production of oceans (Jardillier et al., 2010), with a projected increase of 14% in the intertropical regions over the next 100 years (Flombaum et al., 2013). But their significance is not limited to the global scale of their impact: mounting evidence suggests that rare microbial species can have disproportionate roles in biogeochemical cycles and microbiome functions (Jousset et al., 2017). While at a broader level, *Synechococcus* appears as a physiologically plastic, generalist genus, the increased resolution made possible by improving genomic techniques allows us to see *Synechococcus* as a complex suite of specialists that can cover narrow niches (Farrant et al., 2016).

Thus, understanding *Synechococcus*’ ecological role, and better characterizing phylotype-specific spatial and vertical distribution in an environment like the Black Sea promises to shed light on the dynamics of this important cyanobacterial group.

Expanding on earlier studies of the distribution, abundance, and dynamics of *Synechococcus* in the Black Sea, which concentrated on the euphotic layer (Uysal, 2000, 2001, 2006; Feyzioglu et al., 2004, 2015), we investigated the correlation of total *Synechococcus* counts with environmental variables also deeper in the water column. To increase the resolution of our analysis, we also compared these results to phylotype-specific abundances, distribution patterns, and genetic makeup of two different sets of Black Sea strains. During different field campaigns, we successfully isolated four pure monoclonal *Synechococcus* strains from the western part of the Black Sea: two from an off-shore mesopelagic station (strains BS55D and BS56D) – previously reported (Callieri et al., 2019) – and two new strains (BSA11S and BSF8S) – first described here – isolated from a coastal epipelagic station (referred to as BSD strains and BSS strains, based on their original isolation sources at 750 m off-shore mesopelagic and 6.5 m coastal epipelagic, respectively). We sequenced and compared their genomes to predict their potential role in the Black Sea microbial ecosystem. These data could provide a proxy for their plasticity and adaptation to different environmental conditions, and allow us to understand whether the strains are strictly adapted to specific conditions or their physiological plasticity allows them to inhabit different environments. Moreover, we designed phylotype-specific primers targeting *rpoC1* genes since these sequences provide a robust genetic marker for *Synechococcus* diversity in several marine environments (Palenik, 1994; Tai and Palenik, 2009). In order to better characterize these phylotypes’ distribution and abundance, we also performed qPCR assays on DNA extracted from various Black Sea samples from two vertical profiles in the western and eastern deep part of the Black Sea and in coastal epipelagic sites. Finally, we tested the abundance of these phylotypes also in metagenomes from two stations at different depths.

## Materials and Methods

### Study Site, Sampling Activity, and Chlorophyll-*a* Measurement

**FIGURE 1:**
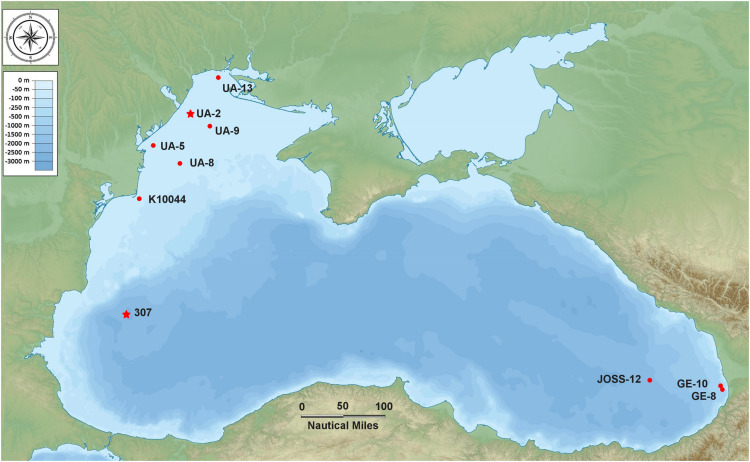
Map of the Black Sea indicating the 10 sampling stations (the two stars indicate the stations where the strains BSD and BSS were isolated).

Seawater samples (*N* = 23) were collected during two successive cruises (aboard the R/V *Mare Nigrum* and the R/V *Akademik*) at 10 sampling stations located in the north-western, western and south-eastern part of the Black Sea from 17 May to 22 June 2016 (Figure 1 and Supplementary Table S1). Water samples throughout the whole column were collected at discrete depths with a 12-Go Flo bottle CTD rosette sampler system (SBE-911 CTD) outfitted with a fluorometer for measuring *in situ* fluorescent profiles. At the epipelagic coastal stations, only surface samples (Surface Homogeneous Layer) were collected, with the exception of three stations (K10044, GE-8, GE-10) where an additional sample was taken at the thermocline. At the two mesopelagic (off-shore) stations samples were collected as follows: at station 307: 25, 42, 110, 500, 750, 1000 m; at station JOSS-12: 12.3, 31.2, 75, 750, 1007 m (including the thermocline, the DCM, Deep Chlorophyll Maximum, and the oxycline) (Supplementary Table S1).

For DNA analysis a variable volume of water (1.7–5.0 L), from all sites and depths (except for one sample from station UA-9, for technical reasons), was filtered using 0.22 μm Sterivex^TM^ Filter Units (Merck) and the filters were stored at -20°C. Additional samples for metagenome analyses were collected from St. 301 (5 m) and St. 307 (5, 30, 150, and 750 m) in October 2019. Up to 6.9 L of sea water from each sampling depth were filtered through a series of 20 μm Nylon Net filters (Millipore), 5 μm polycarbonate membrane filters (Millipore), and 0.22 μm Sterivex^TM^ Filter Units (Merck), which were used to sequence the metagenomes. For flow cytometric analyses, 20 ml of water from each sample were preserved with 1% formaldehyde (final concentration), kept at 4°C in the dark and counted within 6 days. Physico-chemical properties of sea water (temperature, salinity, and nutrients – phosphate, silicate, nitrate, nitrite and ammonia) and chlorophyll-*a* were also measured at each station. Temperature and salinity were measured *in situ* with SBE-911 CTD system. Nutrients were analyzed using standard methods (Solorzano, 1969; Grasshoff et al., 1999). For chlorophyll-*a* analysis water samples were filtered through GF/F Whatman filters with vacuum pump (Millipore) at < 0.2 atm pressure. The filters were stored at −20°C until lab analysis. The chlorophyll-*a* retained by the filters was extracted in 90% cold acetone for 18–20 h at + 8°C - +10°C in a refrigerator. After grinding for 1 min, the extract was centrifuged (ALC, mod. PK 130) at 7000 r/min for 10 min. The spectrophotometric measurements (Nova 400, Merck Spekol 11) were done at the following wavelengths: 750, 665, 663, 645, 630, 480, and 430 nm. After acidification with 1N HCl, within 5 min the extract was measured again at 750 and 665 nm. Three replicates were measured for each sample. The difference between the cuvettes (optical length 5 cm) and the turbidity did not exceed 0.002 at 750 nm. For the calculations the equations of Jeffrey and Humphrey (1975) were used. The precision of the method is 0.1 (mg m^–3^) and the error does not exceed 10% (Edler, 1979).

### Flow Cytometry

The counting was performed using the Flow Cytometer Accuri C6 (Becton Dickinson, Oxford, UK), equipped with a 20 mW 488 nm Solid State Blue Laser and a 14.7 mW 640 nm Diode Red Laser, and 4 fluorescence emission channels. Light scattering signals (forward scatter FSC and side scatter SSC), green fluorescence (FL1 channel = 533/30 nm), orange fluorescence (FL2 channel = 585/40 nm), and red fluorescence (FL3 channel > 670 nm and FL4 channel 675/25 nm) were acquired to discriminate the distinct microbial groups. The counting was done using density plot of FL2-H vs. FL3-H. All data were acquired at a pre-set flow rate of 35 μL min^–1^, keeping the number of total events below 1000 per second. The BD Accuri C6 software (v. 1.0.264.21) was used for data processing.

Bacteria were counted by staining the samples with SYBR Green I (1:10,000 final concentration; Molecular Probes, Thermo-Invitrogen). Density plots of FSC vs. FL1 allowed for optimal distinction between the stained microbial cells and background noise, with a threshold value of 1000 applied on the FL1-H channel and 500 on FSC-H channel.

During strain isolation, an epifluorescence microscope (Zeiss Axioplan) equipped with an HBO 100 W lamp, a Neofluar 100 × objective plus 1.25 × additional magnification and filter sets for blue (BP450-490, FT510, LP520) and green light excitation (LP510-560, FT580, LP590), was used to check the state of the cultures. To calibrate the automatic data acquisition and to validate cell counts, random samples were checked at the epifluorescence microscope (Zeiss Axioplan, Germany).

### *Synechococcus* spp. Strain Isolation

*Synechococcus* strains BS55D and BS56D were previously isolated from samples collected from 750 m at station 307 on 25 June and 28 July 2015 (Callieri et al., 2019), and are here referred to as mesopelagic off-shore BSD (Black Sea Deep) strains. In another field campaign on 17 May 2016 samples were collected from surface coastal water at station UA-2, from which the *Synechococcus* strains BSF8S and BSA11S were isolated, referred to here as epipelagic coastal BSS (Black Sea Surface) strains.

The detailed description of the isolation protocol was published in Callieri et al. (2019). The cells concentrated on a polycarbonate filter were incubated in BG11 prepared using 750 m Black Sea water, 0.2 μm filtered and sterilized. The use of the antibiotic cycloheximide (inhibition of peptide synthesis in eukaryotes) helped to eliminate eukaryotes from the enrichment, and the final purification to obtain a monoclonal strain was performed using flow cytometric single cell sorting with an InFlux V-GS flow cytometer (Becton Dickinson Inc., New Jersey, US) (Callieri et al., 2013). The vials were placed in an incubator held at 20°C and low light (10–15 μmol photons m^–2^ s^–1^).

### DNA Extraction of *Synechococcus* Cells From Field Samples and From Strains

The Sterivex^TM^ filters (22 out of 23) from the field samples were cut into pieces and the DNA extraction was performed in triplicate using PowerSoil^®^ DNA Isolation Kit (Qiagen) according to the manufacturer’s instruction. The same protocol was applied for the DNA extraction from the pelleted cultured strains (BSA11S and BSF8S). DNA from metagenomic samples was extracted with phenol-chloroform methodology (Martín-Cuadrado et al., 2007). Filters were treated with CTAB lysis buffer and then with 1 mg ml^–1^ lysozyme and 0.2 mg ml^–1^ proteinase K (final concentrations) prior to the phenol/chloroform/isoamyl alcohol and the chloroform/isoamyl alcohol steps.

### Sequencing, Assembly, Annotation, Genomic Comparison, and Phylogenomics of Black Sea Strains and Metagenomes

Black Sea *Synechococcus* sp. BS55D and BS56D (BSD strains) were previously sequenced and analyzed (Callieri et al., 2019). The cultures were pure monoclonal, not axenic cultures. Here we introduce two new strains isolated from surface (BSA11S and BSF8S) Black Sea waters; their DNA was extracted and sequenced as described previously for BSD strains (Callieri et al., 2019). Black Sea metagenomic samples were sequenced in an entire lane of Illumina HiSeq X Ten PE 2 × 150 bp (Novogene company), which provided 20 Gb of output for each sample.

All four strains analyzed here are draft genomes obtained in several contigs (42 and 44 contigs for BSA11S and BSF8S, respectively; 7 and 8 contigs for BS55D and BS56D, respectively), but their genomes are 100% complete assessed with *Synechococcus* specific markers with CheckM (Parks et al., 2015). Assembly of BSS strains was conducted with SPAdes (Bankevich et al., 2012) following careful, only-assembler and default k-mer parameters. Gene prediction was conducted with PRODIGAL (Hyatt et al., 2010). Annotation of CDS was done with BLAST (Altschul et al., 1997) and RAST (Overbeek et al., 2013), KEGG KO (Kanehisa et al., 2016), COG (Tatusov et al., 2001), and TIGR (Haft et al., 2001) databases. Genomic analysis was performed using the abovementioned tools and average nucleotide identities (ANI) between strains were calculated as described elsewhere (Goris et al., 2007). Core and flexible genes of Black Sea strains were determined with the GET_HOMOLOGUES software (Contreras-Moreira and Vinuesa, 2013) and their core/flexible genome was solely calculated among themselves. A maximum-likelihood phylogenomic tree with 396 universal markers and > 100 genomes of *Synechococcus*, *Prochlorococcus*, and *Cyanobium* from marine, brackish, euryhaline and freshwater habitats was built with PhyloPhlAn tool (Segata et al., 2013).

### Primer Design and *Synechococcus* Phylotype Quantification by qPCR

The genomic sequences of the strains (BSD and BSS) were used to design the primers to quantify their specific *rpoC1* gene, in order to estimate the difference in their abundance for all collected seawater samples. Sequences of the *rpoC1* gene from all four strains were aligned using MUSCLE (Edgar, 2004) with the online multiple sequence aligner provided by EMBL-EBI^1^. Since the similarity between the *rpoC1* sequences of the two BSD strains was 99%, and 100% between the BSS ones, only two primer pairs were designed, one for each set. Primers were designed specifically for the *rpoC1* sequence of the strains with the goal to amplify fragments of around 100 bp and with a theoretical annealing temperature of around 60°C. Primers were designed using NCBI Primer BLAST (Ye et al., 2012) or Primer 3 (Untergasser et al., 2012) and target specificity was checked by BLAST. The chosen primers for the BSD strains were 816F- CACCTCTGACCTCAACGACC and 935R- TCCTGGAGCATCCGCTTTTC, and for the BSS strains 472F- GACCTCACCTACAAGCAACTCC and 570R-CACCTCAGGCTCGTTTTCTATC. None of the primer pairs had hits with 100%, thus the *in silico* specificity of the primer pairs was very high. Hits by single primers with 100% accuracy are summarized in Supplementary Table S2: only one match was a *Synechococcus rpoC1* gene (primer 427F, non-target sequence accession number AF448108) and the sequences did not match the reverse primer. The annealing temperature of the primers was determined through a gradient PCR with annealing temperatures between 56 and 63°C (8 steps). This was done using the GoTaq GreenMasterMix (Promega) in 25 μl according to manufacturer’s instructions, the DNA of the strains, and the following protocol: initiation 95°C 1′, 30 times denaturation 95°C 0.5′, annealing 0.5′, elongation 72°C 1′, and the final extension 72°C 7′. Amplicons were visualized by agarose gel electrophoresis and an annealing temperature of 62°C was chosen for both primer pairs.

All DNA samples extracted from seawater were twofold diluted and tested for the abundance of *rpoC1* genes using the two primers pairs. The qPCR assays were carried out using the RT-thermocycler CFX Connect (Bio-Rad Laboratories Inc., United States). qPCRs were performed in a volume of 20 μl containing 1XSsoAdvanced^TM^ Universal SYBR^®^ Green Supermix (Bio-Rad), 0.5 μM of each primer, 2 μl template DNA and autoclaved MilliQ water (Millipore) to the final volume. The program of qPCR was 95°C for 2 min, 35 cycles of 95°C for 15 s, 62°C for 30 s, and 72°C for 15 s. In order to evaluate the specificity of reaction, the melting curve analysis was performed from 60 to 95°C with increments of 0.5°C 5 s^–1^ and the amplicon size was verified by gel electrophoresis (an example of electrophoresis gel picture is showed in Supplementary Figure S1). The standard curves were done by a 10-fold dilution of the purified and quantified amplicon for each target as previously described (Di Cesare et al., 2013). The mean value and the standard deviation of the qPCR efficiency were 97.25 ± 6% and *R*^2^-values were always > 0.99. The limits of quantification (LOQ) were determined as previously described (Bustin et al., 2009), they were 11 and 23 copies μl^–1^ of the *rpoC1* genes in the BSD and the BSS strains, respectively.

### Recruitment Analysis of *Synechococcus* Phylotypes on Black Sea Metagenomes

To estimate the relative abundance of *Synechococcus* genomes we performed read recruitment and mapping, which was assessed considering BLASTN hits of the genome into the metagenomic reads at > 70% identity and > 50 bp of alignment length. Reads mapping at > 95% of identity were considered as belonging to the same *Synechococcus* species. Reads per Kb of genome per Gb of metagenome (RPKGs) at > 95% identity and > 50 bp of alignment length were also calculated for each genome on the different metagenomes.

### Statistical Analyses

All statistics were conducted on 22 samples using the software R version 3.51. To see how similar samples were in terms of their physical and chemical properties Bray-Curtis dissimilarity was calculated using the data for station depth, temperature, salinity, PO_4_, NH_4_, and Si using vegan package version 2.5-6 (Oksanen et al., 2007). Clustering was depicted using hclust with average linkage of the samples. We tested how much of the variance was explained by the station characteristics (epipelagic coastal / epipelagic off-shore / mesopelagic off-shore, see Supplementary Table S3) and the sampling depth using PERMANOVA analysis (*adonis* command in vegan).

Statistical evaluation of the abundances of *Synechococcus* (flow cytometry and qPCR counts) and bacteria (flow cytometry counts) was done using two sets of models: (1) Related to the physical location of the organisms using the factors: sampling depth and the characterization of the station as epipelagic coastal / epipelagic off-shore / mesopelagic off-shore (model: abundance ∼ sampling depth + station characteristics). (2) Related to chemical and physical variables measured. For the latter, correlations between variables were evaluated using Pearson’s moment correlation and temperature, salinity, NH_4_ (correlated with PO_4_; *r* = 0.95), and Chl-*a* were chosen as variables for the models (model: abundance ∼ Temp + Salinity + NH_4_ + Chla). Models of set 1 and 2 are not independent since all chemical/physical variables tested varied significantly with station characteristics and station depth.

For *rpoC1* of the BSD phylotype both occurrence (presence/absence data) and abundance (genes ml^–1^) were evaluated, whereas for *rpoC1* of the BSS phylotype only presence/absence data was used. Linear models (lm) of log-transformed data were performed in all cases of abundance data (linear regression model), whereas for presence/absence data generalized mixed models (glm) with a binomial distribution of the data were conducted. The models were evaluated visually by plotting the residuals in order to find a model with the best fit. The results of these models were depicted in an ANOVA like manner using the ANOVA function from the package *car* (Version 3.0-8) (Fox and Weisberg, 2019).

### Data Availability

BSD Black Sea *Synechococcus* genomes were previously published (Callieri et al., 2019) and are available in the NCBI-GenBank databases under the BioProject PRJNA419515. The novel coastal BSS strains are available under the BioProject number PRJNA556564. *Synechococcus* sp. BSA11S is codified by BioSample SAMN12359518 and GenBank accession number VNWP00000000. *Synechococcus* sp. BSF8S is codified by BioSample SAMN12359523 and GenBank accession number VOBS00000000. Black Sea metagenomic datasets were deposited under the NCBI database under the BioProject PRJNA638805.

## Results

### Environmental Factors

In the Surface Homogeneous Layer the temperature (T) ranged between 13.31 and 17.59°C and salinity (S) from 7.00 to 18.15 PSU, whereas in deep waters (500–1007 m) both T (8.9–9.04) and S (22.04–22.28) were almost constant (Supplementary Table S1). Concentrations of nutrients fluctuated, ranging from 0 to 204 μg L^–1^ for PO_4_; 0.5–2490 μg L^–1^ for NO_3_; 0–10 μg L^–1^ for NO_2_; 0–760 μg L^−1^ for NH_4_; and between 12 and 7500 μg L^–1^ for Si (Supplementary Table S1). Vertical profiles of phosphate and ammonia revealed higher values at station 307 compared to JOSS-12, while the opposite was observed for nitrate and silicon.

Surface chlorophyll-*a* (Chl-*a*) ranged from 1.84 (st. GE-8) to 14.30 μg L^–1^ (st. UA-5) and in general, higher Chl-*a* values were detected at coastal stations. The vertical distribution showed a Chl-*a* maximum at 31–42 m depth in the mesopelagic stations (0.79 μg L^–1^ at st. JOSS-12, and 0.55 μg L^–1^ at st. 307) (Supplementary Table S1). In contrast with previous observations, Chl-*a* concentrations in the deep water layers (below 75 m) were below detection limit of the method used.

Differences between the 22 analyzed samples were characterized using the Bray-Curtis distance, based on physical and chemical properties (station depth, temperature, salinity, PO_4_, NH_4_, Si). Permutational analysis of variance of the distance matrix (PERMANOVA) showed that 67% of variance between sampling points was explained by the station characteristics (epipelagic coastal / epipelagic off-shore / mesopelagic off-shore) and only 3% by the sampling depth. Thus, the different sampling sites are clearly grouped by their geographic location (Supplementary Figure S2 and Supplementary Table S4).

### Total *Synechococcus* spp. and Bacterial Abundance by Flow Cytometry

**FIGURE 2:**
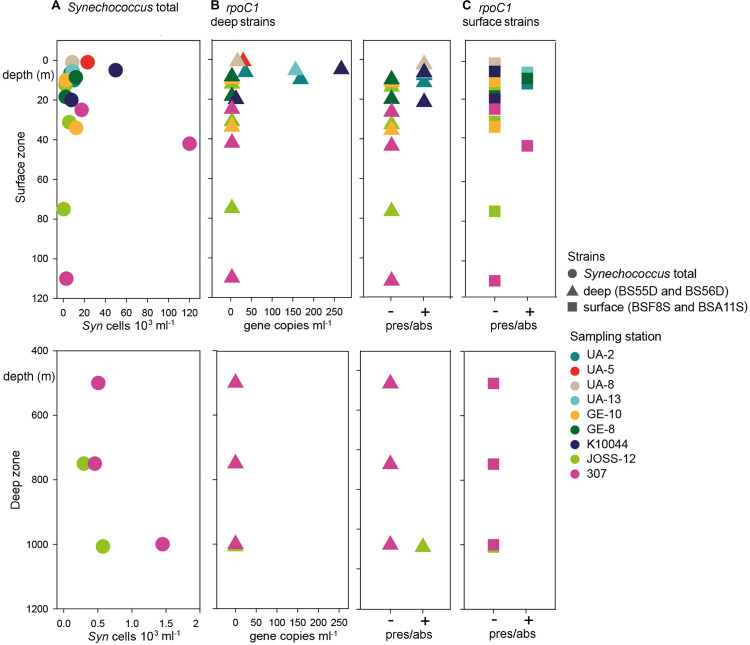
Abundance of *Synechoccocus* in the Black Sea. Upper panels: epipelagic zone between 0 and 120 m depth. Lower panels: Deep zone between 400 and 1200 m depth. **(A)** *Synechococcus* (*Syn*) cell abundance as determined by flow cytometry. Note that both *X*-axes are scaled differently in the upper and lower panels. **(B)** Left: abundance of *rpoC1* gene copies affiliated with BSD strains BS55D and BS56D; Right: presence/absence of genes as determined by qPCR. **(C)** Occurrence of *rpoC1* genes affiliated with BSS strains BSF8S and BSA11S. Colors code for the sampling stations and shapes for the type of data.

Flow cytometry data showed the presence of *Synechococcus* in all stations and at all depths sampled, including the deep layers down to 1007 m. Total *Synechococcus* cell abundances ranged from 3.9 × 10^2^ to 1.2 × 10^5^ cells ml^–1^ in the zone from surface to 100 m (halocline), and from 2.9 × 10^2^ to 1.4 × 10^3^ cells ml^–1^ in deep waters (500–1007 m depth) (Figure 2). The peak of abundance was at the deep chlorophyll maximum (42 m at st. 307). *Synechococcus* abundances were not significantly correlated to the station characteristics nor to the sampling depth (Table 1). Similarly, no difference was found between chemical and physical properties and *Synechococcus* abundances (Table 2).

**TABLE 1:** Statistical results for the influence of station characteristics (epipelagic coastal, epipelagic off-shore, mesopelagic off-shore) and sampling depth on the abundance (lm) and occurrence (glm) of bacteria and *Synechococcus* spp. BSD (Black Sea Deep) phylotype, BSS (Black Sea Surface) phylotype.

Lm	Estimate	*df*	*t*-value	*p*-value
**Bacteria**				
Sampling depth	0.112	1	0.328	0.574
Station characteristics	3.510	2	5.131	0.017*
***Synechococcus* spp.**				
Sampling depth	1.073	1	0.669	0.424
Station characteristics	2.531	2	0.788	0.470
**Quantity BSD**				
Sampling depth	0.024	1	0.009	0.926
Station characteristics	23.264	2	4.228	0.031*

**Glm**	**Chi^2^**	***df***		***p*-value**

**Occurrence BSD**				
Sampling depth	1.859	1.000		0.173
Station characteristics	10.316	2.000		0.006**
**Occurrence BSS**				
Sampling depth	0.118	1.000		0.731
Station characteristics	0.167	2.000		0.920

*p < 0.05, **p < 0.01.

**TABLE 2:** Statistical results for the linear models on the influence of chemical and physical conditions on the abundance (lm) and occurrence (glm) of bacteria and *Synechococcus* spp. BSD (Black Sea Deep) phylotype, BSS (Black Sea Surface) phylotype.

	Estimate	±	*t*-value	*p*-value
**Bacteria**				
Temperature	0.076	0.031	2.482	0.024*
Salinity	–0.084	0.035	–2.386	0.029*
Ammonia	0.000	0.000	0.144	0.888
Chlorophyll-a	0.100	0.031	3.223	0.005**
***Synechococcus* spp.**				
Temperature	–0.020	0.109	–0.181	0.858
Salinity	–0.163	0.125	–1.305	0.209
Ammonia	–0.002	0.002	–1.004	0.330
Chlorophyll-a	0.115	0.111	1.042	0.312
**Quantity BSD**				
Temperature	–0.055	0.111	–0.498	0.625
Salinity	–0.353	0.128	–2.755	0.014*
Ammonia	0.001	0.002	0.510	0.617
Chlorophyll-a	0.184	0.113	1.625	0.123
**Occurrence BDS**				
Temperature	–0.15	0.24	–0.60	0.547
Salinity	–0.24	0.26	–0.95	0.344
Ammonia	–0.01	0.05	–0.19	0.851
Chlorophyll-a	1.43	1.02	1.40	0.161
**Occurrence BSS**				
Temperature	–0.349	0.312	–1.116	0.264
Salinity	–0.287	0.257	–1.119	0.263
Ammonia	–0.010	0.065	–0.159	0.874
Chlorophyll-a	1.698	1.045	1.625	0.104

*p < 0.05, **p < 0.01.

We also quantified the total prokaryotic cell abundance (excluding *Synechococcus*), which ranged from a minimum of 1.5 × 10^5^ to a maximum of 2.1 × 10^6^ cells ml^–1^ reached in epipelagic waters (0–20 m depth) (Supplementary Table S1). Bacteria were significantly different between the sampling stations with different characteristics (epipelagic coastal / epipelagic off-shore / mesopelagic off-shore), however, no significant difference was found for depth (Table 1). Bacterial abundances were significantly positively influenced by Chl-*a*, and slightly influenced by temperature, or salinity, but not by NH_4_ (Table 2).

### Phylotype-Specific *Synechococcus* Presence and Abundance

Through qPCR analysis we found BSD phylotypes more frequently than BSS ones. BSD were detected in low abundance in 36% of samples (95 *rpoC1* gene copies ml^–1^ average concentration, Figure 2 and Supplementary Table S1), mainly in the northwest part of the Black Sea with higher abundances in surface waters (up to 269 *rpoC1* gene copies ml^–1^ in 5 m depth). Interestingly, at station JOSS-12 these strains were present only in the sample from the deepest layer (1007 m depth), even if in low abundance (below LOQ).

BSS phylotypes occurred rarely and in very low abundance (below LOQ in most cases). They were detected by qPCR only in the upper water layers across the sampling sites. The maximum depth where BSS were detected was 42 m at station 307. In 14% of all samples, BSS and BSD phylotypes co-occurred (Figure 2 and Supplementary Table S1).

The total abundances of *Synechococcus* cells and the abundances of *rpoC1* genes of BSD (the only set for which abundance was tested) were not correlated (Pearson’s coefficient 0.21). BSD was influenced by the station characteristics in terms of quantity and presence/absence, which was not the case for BSS (Table 1). Presence/absence of both tested *rpoC1* genes was not related to any chemical/environmental factor tested, whereas the quantity of BSD related *rpoC1* was negatively influenced by salinity (Table 2).

**FIGURE 3:**
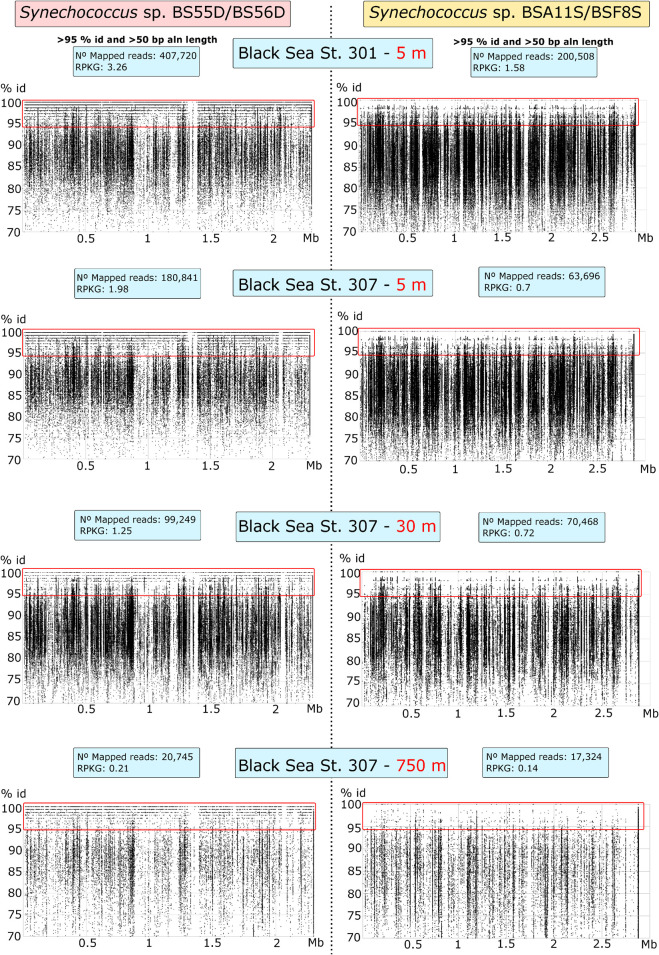
Recruitment plots of *Synechococcus* sp. BS55D/BS56D and BSA11S/BSF8S phylotypes on Black Sea metagenomic datasets from stations St. 301 (5 m) and St. 307 (5, 30, and 750 m). Each dot represents a single read of the metagenome mapped into the genome of each phylotype. Reads mapped at > 70% of identity and > 50 bp of alignment length with BLASTN are shown. *X*-axis represents the position of the genome (Mb) and *Y*-axis the % of identity. Species level threshold to assess presence was set up to > 95% of identity reads and was shown inside the red rectangle. Left panels show recruitment plots of BS55D/BS56D phylotypes and right panels show recruitment plots of BSA11S/BSF8S phylotypes on the metagenomes. Blue boxes show total mapped reads and Reads per Kb of genome per Gb of metagenome (RPKGs) for each genome and metagenome at > 95% of identity and > 50 bp of alignment lengths.

We also tested the presence of Black Sea phylotypes in five different sample depths: 5 m (St. 301) and 5, 30, 150, and 750 m (St. 307). We mapped the metagenomic reads with the *Synechococcus* genomes with BLASTN and showed recruitment plots at > 70% identity and > 50 bp of read-genome alignment lengths (Figure 3). We assessed the presence of any strain when reads mapped at > 95% of identity throughout the genome. All BLASTN reads with identities between 70 and 95% were related to other *Synechococcus* spp. Our results show that BS56D/BS55D phylotypes were found in both stations at the epipelagic layer of 5 m and were significantly less abundant with the increasing depth, but still detectable both at 30 m and in very low numbers at 750 m, which correlates well with the abovementioned qPCR results. Even if in very low numbers, we must highlight the presence of various reads mapping into the original deep isolate at 750 m. Nonetheless, it is evident that the preferred habitat for this strain was the epipelagic and photic layer, as the phylotype was rare in euxinic waters.

On the other hand, BSA11S/BSF8S phylotypes were not detected above the species level in any of the metagenomes, although similar species clearly inhabit the ecosystem as seen in the recruitment plots > 90% identity.

### Phenotypic and Genomic Features of *Synechococcus* Strains

The main characteristics of the BSD strains were detailed previously (Callieri et al., 2019). The newly described epipelagic coastal strains (BSA11S and BSF8S) were isolated from near-surface samples (6.5 m depth) taken at the coastal station UA-2 in May 2016. They were phenotypically similar to the BSD strains, appearing pink in culture with a peak of absorption of phycoerythrin at 573 nm and of Chl-*a* at 443 and 682 nm, and with a low peak of allophycocyanin at 643 nm, hardly distinguishable from Chl-*a* (Supplementary Figure S3).

**FIGURE 4:**
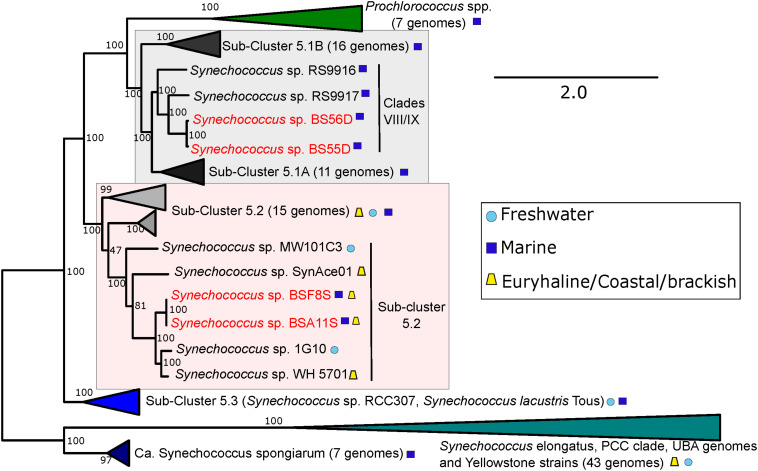
Phylogenomic tree of BSS (BSA11S and BSF8S) and BSD (BS55D and BS56D) phylotypes constructed with PhyloPhlan using 396 universal genes. 100 bootstraps were calculated. Selected marine, freshwater and brackish/euryhaline/coastal strains and clades are displayed. Black Sea strains are colored in red, symbols next to strain names indicate their origin.

An updated picocyanobacterial phylogeny is shown in Figure 4, including both BSD and BSS strains. As previously noted, the BSD strains fall inside clades VIII/IX (with closest neighbors RS9917 and RS9916) of the marine sub-cluster 5.1A (Callieri et al., 2019). The new BSS strains (BSA11S and BSF8S) branch inside the polyphyletic and diverse (in terms of genomic distance and habitats of origin) sub-cluster 5.2, with the euryhaline Long Island Sound strain WH5701 as closest relative (Dufresne et al., 2008).

**FIGURE 5:**
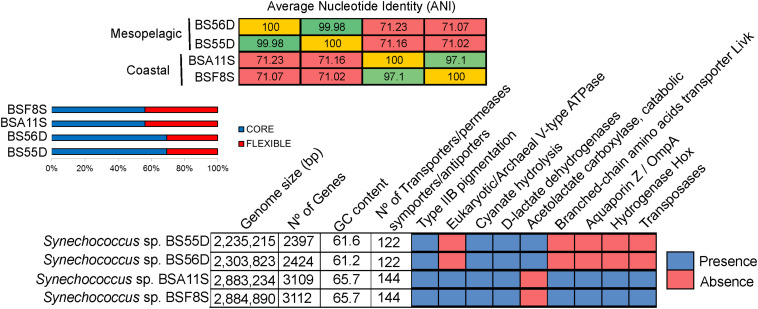
Average Nucleotide Identity (ANI), pan-genome analysis and genomic features of BSS (BSA11S and BSF8S) and BSD (BS55D and BS56D) Black Sea strains.

At the genomic level, the strains differed considerably (Figure 5). BSS strains displayed larger genomes (2.88 Mb) and higher GC content (65.7%) compared to BSD strains (2.25–2.3 Mb genome size and 61% GC content). The two BSS *Synechococcus* strains exhibit a very high Average Nucleotide Identity (ANI = 99.98%) and can be thus considered the same species. The same holds true for BSD strains (displaying > 97% of ANI, same species), while both sets of strains only share ca. 71% of ANI and can be thus considered as different genera. BSS and BSD strains contain 43.9% and 30.7% of unique genes, respectively. The relatively high percentages (55–69%) of shared genes (core genome) between all four Black Sea strains is in accordance with what was observed for their closest relatives *Synechococcus* sp. RS9917 (a halotolerant strain related to BSD strains) (Fuller et al., 2003) and WH5701 (closely related to BSS strains) (Dufresne et al., 2008).

Looking at the specific genes that were shared and unique among Black Sea strains, we noticed that BSS strains harbor a high number of transposases (*n* = 69). These were located close to each other in the genome, or flanked by different genes such as those encoding phycobilisomes (PBS operon), a V-type ATPase, D-lactate dehydrogenases, copper-resistance protein, cytochrome P450, aquaporin Z, phosphate ABC transporter substrate-binding protein, bidirectional hydrogenase (*hox*) and additional genes (*hyp*), lysozyme/peptidoglycan-binding proteins (common to *Microcystis*), or an ion channel protein (MscS). In contrast, we did not detect any transposases in BSD strains (Figure 5).

Both sets of strains have the genetic potential to perform cyanate hydrolysis to obtain an additional ammonia source and CO_2_. The number of transporters for different nutrients, antiporters/symporters was higher in BSS (*n* = 144) than in BSD strains (*n* = 122). It is noteworthy that BSS representatives contained 11 high light inducible proteins whilst BSD strains showed only 4. On the other hand, only the BSD strains contained genes for the catabolic acetolactate carboxylase (involved in acetoin fermentation), together with the presence of a very specific type of D-lactate dehydrogenase, which was so far described only for strain RS9917 and no other picocyanobacteria (Callieri et al., 2019). All four strains show type IIB pink pigmentation, however, the structure of the Phycobilisome (PBS) operon differed, as BSD strains possess one additional subunit of phycocyanin while BSS strains contain a transposase along with the PBS cluster (Supplementary Figure S4).

## Discussion

### *Synechococcus* spp. Dynamics and Its Correlation With Environmental Factors

*Synechococcus* cell numbers were higher in the Black Sea’s surface water, in agreement with the distribution of autotrophic picoplankton cells in the oceans (Scanlan, 2012). This result was also in agreement with epifluorescence microscopy counts, where total *Synechococcus* abundances in the Black Sea ranged between 10^2^ and 10^5^ cell ml^–1^ in the first 160 m (Uysal, 2006). The detection of *Synechococcus* in oxygenated mesopelagic waters was reported previously (Vilibić and Šantić, 2008; Sohrin et al., 2011). However, their vertical and horizontal distribution in a deep meromictic system like the Black Sea was so far never investigated and, to our knowledge, no information was available on their correlation with environmental factors.

We found that the abundance of *Synechococcus* cells was not correlated to bacterial abundance (Pearson’s correlation coefficient: 0.16). *Synechococcus* spp. were always detectable, and their numbers generally decreased with depth, and only slightly increased at 1000 m compared to 500 m. Bacterial numbers were not influenced by depth, in contrast with what was previously found in the Sargasso Sea, where water depth was one of the main factors influencing the abundances of both *Synechococcus* and bacterial cells (Sjöstedt et al., 2014). Furthermore, salinity had no discernible impact on total *Synechococcus* cell abundance, while in other studies and years a correlation with salinity in the Black Sea has been reported (Uysal, 2000, 2006). We found that total *Synechococcus* cell numbers, unlike bacteria, were not correlated with Chl-*a*, which mainly derived from the eukaryotic fraction, as previously reported in other marine systems (Tai and Palenik, 2009).

BSD phylotypes were detected more frequently than BSS ones, and they were mostly found in 5 m metagenomes and epipelagic samples tested with qPCR, showing that they are species with preferred epipelagic habitats. However, even if at low numbers, they were also detected at 750 and 1007 m depth, both with metagenomic recruitment and qPCR techniques, confirming their presence and supporting the suggested adaptability of BSD to those depths (Callieri et al., 2019). The abundance of BSD phylotypes (by qPCR) was higher in coastal sites than in off-shore epipelagic ones, and it was negatively influenced by salinity, suggesting that these two factors could be among the main drivers of the distribution of this particular phylotype within the variables that have been analyzed.

### Genomic Comparison of Coastal and Pelagic *Synechococcus* Strains

Comparative genomics of coastal and pelagic strains made it possible to characterize, along with the core genome of *Synechococcus*, also the flexible genes that diverge between strains (Dufresne et al., 2008) and thus provide a higher resolution of *Synechococcus* dynamics in the Black Sea. An important feature unique to BSS was the presence of archaeal/eukaryotic V-type ATPases in their genomes. To our knowledge, these genes have only been observed in strains of *Synechococcus* sp. WH5701, BO8801, PCC7336, and *Cyanobium* sp. PCC7001. In fact, the closest organisms harboring these V-type ATPases are other filamentous Cyanobacteria (*Leptolyngbya* or *Cyanothece*) and different Gammaproteobacteria (Methylococcaceae) and Alphaproteobacteria (Rhodospirillaceae). The presence of V-type ATPases in epipelagic coastal strains (in addition to two operons with the typical F0F1 ATPases) remains enigmatic. Another unique feature of the BSS was the presence of a bidirectional hydrogenase (*hox*) and additional genes (*hyp*), putatively involved in the uptake and photoproduction of molecular hydrogen (Tamagnini et al., 2002). Hydrogenases are widespread in anoxygenic photosynthesizers and other filamentous N-fixing Cyanobacteria (Ludwig et al., 2006), but have recently been observed in planktonic freshwater picocyanobacteria (Di Cesare et al., 2018; Cabello-Yeves et al., 2018). Hence, it appears that the BSS phylotypes have many different mechanisms to produce and incorporate H^+^. These strains also contain genes annotated as D-lactate dehydrogenases, although of a different origin from those present in pelagic strains, which are found in some other Cyanobacteria and Proteobacteria.

Importantly, both BSD and BSS strains display type IIB pigmentation (Supplementary Figure S3), a novel type of pigmentation that was first observed in freshwater and brackish picocyanobacteria (Larsson et al., 2014; Cabello-Yeves et al., 2018; Sánchez-Baracaldo et al., 2019), and then described in marine strains (Callieri et al., 2019). Furthermore, the fact that the above-mentioned unique genes were flanked by transposases in BSS genomes could indicate that these were transferred horizontally by other cyanobacteria, and were probably involved in the niche adaptation of the two coastal strains. Indeed, genes of the phycobilisome operon flanked by mobile genetic elements were most similar to the ones from freshwater strains from sub-cluster 5.2, which once again opens up new evolutionary perspectives on the freshwater to marine transition suggested for this picocyanobacterial sub-cluster (Sánchez-Baracaldo, 2015; Sánchez-Baracaldo et al., 2019). In general, all genes encoded in the flexible genome have been linked to genomic islands, previously associated with niche adaptation in marine and freshwater picocyanobacteria (Dufresne et al., 2008; Scanlan et al., 2009; Cabello-Yeves et al., 2018). Overall, based on the genomic features observed in our Black Sea strains, we could conclude that BSS phylotypes seem to be more adapted to surface layers, as they were only detected in the upper layers of some Black Sea stations by qPCR (except for one station at 42 m) and were rare from the metagenomes. They also contain more high light inducible proteins or transporters (including a V-type ATPase, an aquaporin Z, and hydrogenases), which suggests that they may be specialists of the photic layers of the Black Sea. On the other hand, BSD phylotypes have evolved together within the marine sub-cluster 5.1 (Scanlan et al., 2009). These phylotypes were detected in surface, epi- and mesopelagic layers (including 750 and 1000 m) and hence could be adapted to variable salinities by being halotolerant, just as their closest relatives from the Red Sea (Fuller et al., 2003). However, we must clearly highlight that their preferred habitat were surface and epipelagic waters, as confirmed by recruitment analysis. Nonetheless, their detection in mesopelagic off-shore euxinic waters, even in low numbers (around 1000 cells ml^–1^) as previously discussed, appears significant, and possibly correlated with a role in the “deep red fluorescence” Chl-*a* signal characteristic of the deep mesopelagic waters of the Black Sea (Callieri et al., 2019). The putative capability of BSD strains to perform heterolactic and acetoin fermentations remains to be confirmed, and further *in vitro* experiments will test if the mere presence of these genes allows them to perform this metabolism.

## Conclusion

The total abundance and distribution of *Synechococcus* in the Black Sea did not correlate significantly to most key environmental variables we tested. This suggests that the high genomic variability of this cyanobacterial genus informs more textured and complex strain-specific dynamics that deserve further attention. Providing us with a higher resolution, whole genome sequencing and qPCR techniques indicate that: (1) BSD phylotypes could reach deep waters from the upper epipelagic layers, and are able to withstand the aphotic and oxygen depleted conditions of deep layers, displaying higher resilience and adaptably, while, (2) BSS phylotypes seem to be restricted to epipelagic waters, as specialists of this photic niche.

These results contribute to the growing definition of *Synechococcus* dynamics and phylogenetics, especially in the unique euxinic conditions of the Black Sea. Already identified as an important analog for the Proterozoic ocean, this is a crucial environment to shed light on the oxygenation of the planet, along with its enormous impact on evolution, and its significance for contemporary climate change. As the importance of the rare biosphere becomes apparent, we need better resolved analyses of the distribution, abundance, dynamics, and evolution of microbial populations, and the higher resolution of *Synechococcus* dynamics in the Black Sea provided here is a step in this direction.

## Data Availability Statement

The BSD Black Sea *Synechococcus* genomes were previously published (Callieri et al., 2019) and are available in the NCBI-GenBank databases under the BioProject PRJNA419515. The novel coastal BSS strains are available under the BioProject number PRJNA556564. *Synechococcus* sp. BSA11S is codified by BioSample SAMN12359518 and GenBank accession number VNWP00000000. *Synechococcus* sp. BSF8S is codified by BioSample SAMN12359523 and GenBank accession number VOBS00000000. Black Sea metagenomic datasets were deposited under the NCBI database under the BioProject PRJNA638805.

## Author Contributions

CC, AD, PC-Y, ND, and SM conceived the study. AD, PC-Y, ND, CC, FB, and MS wrote the manuscript. CC isolated the *Synechococcus* strains. VS, NS, ND, SM, LK, and EP carried out the samplings and organized the research cruise. MS, PC-Y, FR-V, and EE did the sequencing and analyzed the sequences. AD and ND did the qPCR analyses. GC and CC made the cytometer countings and prepared the samples for sequence analyses. RB provided laboratory support and chemical analyses. All authors commented the manuscript.

## Conflict of Interest

The authors declare that the research was conducted in the absence of any commercial or financial relationships that could be construed as a potential conflict of interest.

## Footnotes

1 https://www.ebi.ac.uk/Tools/msa/muscle/
