# Supplementary material for: Genomic Comparison and Spatial Distribution of Different Synechococcus Phylotypes in the Black Sea
Source: Front Microbiol. 2020 Aug 12;11:1979. doi: 10.3389/fmicb.2020.01979 (PMC7434838; doi:10.3389/fmicb.2020.01979)
Supplement: Supplementary file 1 [file Data_Sheet_1.docx]

**Supplementary Material**


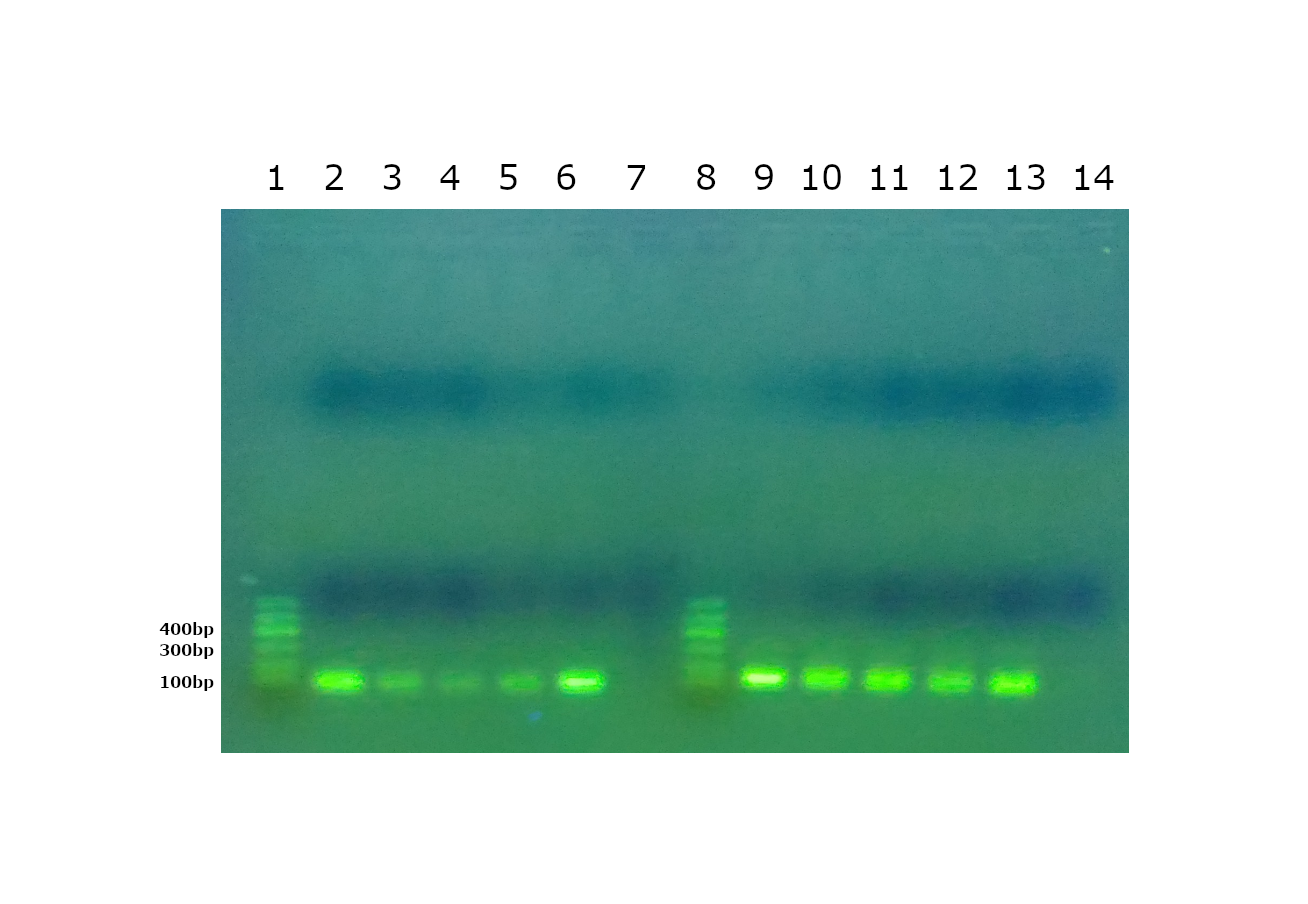


**Fig. S1.** Agarose gel of Real Time PCR amplicons of *rpoC*1 gene, using the primers 472F- GACCTCACCTACAAGCAACTCC and 570R- CACCTCAGGCTCGTTTTCTATC, designed to quantify the coastal strains and the primers 816F217 CACCTCTGACCTCAACGACC and 935R- TCCTGGAGCATCCGCTTTTC, designed to quantify the pelagic strains. In detail, lanes 1and 8: DNA ladders; lanes 2 and 6: DNA from the positive control BSA11S strain; lanes 3, 4 and 5: DNA from positive epipelagic coastal water samples; lane 7: negative control; lanes 9 and 13: DNA from the positive control BS55D strain; lanes 10, 11 and 12: DNA from positive mesopelagic off-shore water samples; lane 14: negative control.


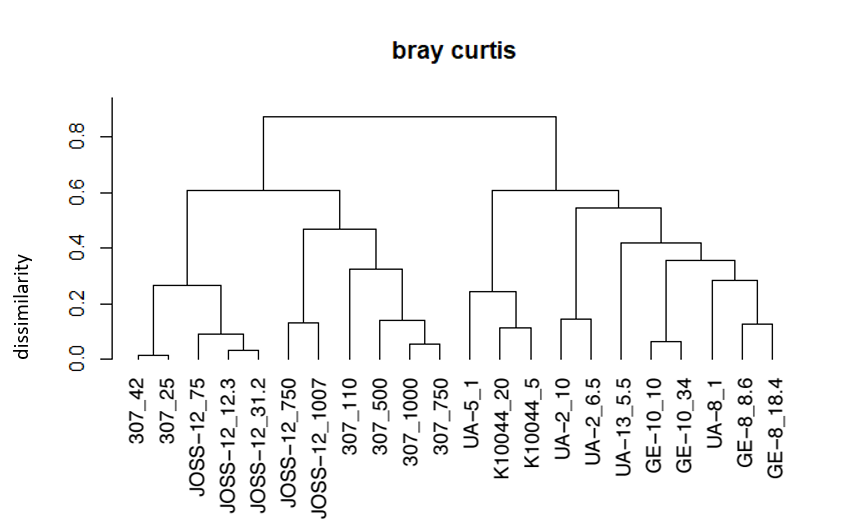


**Fig. S2.** Average linkage clustered dendrogram based on bray curtis dissimilarity of the characterisation of stations based on chemical/physical parameters (station depth, temperature, salinity, PO_4_, NH_4_, Si).


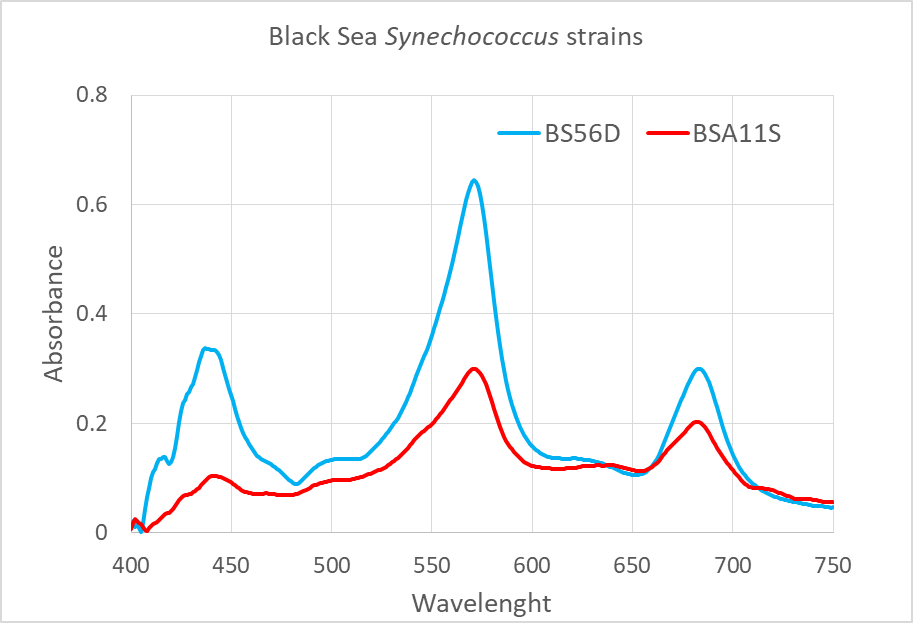


**Fig. S3.** Absorption spectra of the two *Synechococcus* strains BS56D (mesopelagic BSD) and BSA11S (epipelagic coastal BSS). In vivo absorbance spectra of the diluted cultures were measured with a double monochromator spectrophotometer (SAFAS, UVMC2) in the wavelength range 400 – 750 nm. Spectra were recorded with a 1 nm interval and 5 nm slit width in a quartz cuvette of 5 cm. 50µL of NaClO 10% was directly added in the cuvette and the samples were read again to measure the scatter. Absorbance due to scatter was subtracted from the spectra values at each wavelength for each sample.


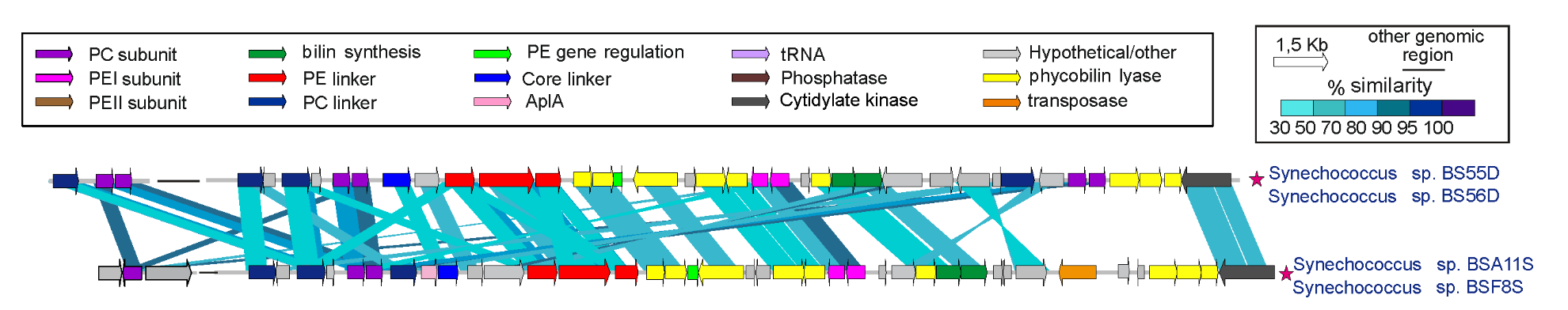


**Fig. S4.** Similarity of the phycobilisome (PBS) operon between epipelagic coastal BSS strains (BSA11S, BSF8S) and mesopelagic BSD strains (BS55D, BS56D). Different subunits are colour-coded and symbolized accordingly with arrows.

**Tab. S1.** Location of the sampling stations of the Black Sea and values of the chemical, physical and biological parameters considered in the statistical analyses and the results of the qPCR of the *rpoC1* gene. Epipelagic coastal BSS (BSA11S, BSF8S) and mesopelagic BSD (BS55D, BS56D). Neg: negative result, NQ: positive but not quantifiable, BDL: below detection limit, NM: not measured.

**Tab. S2.** Hits with 100% of coverage and similarity resulting from BLAST analysis of the designed primer pairs.

| **Primer:** | **Accession Nr.** | **Organism** |
| --- | --- | --- |
| **427F & 570R** | *No hit* |  |
| **870F & 935R** | *No hit* |  |
| **427F** | AF448108 | *Synechococcus* sp. (*rpoC1* gene) |
| **570R** | *No hit* |  |
| **870F** | CP001683.1 | *Saccharomonospora viridis* |
|  | CP031158.1 | *Deinococcus wulumuqiensis* |
|  | CP036295.1 | *Desulfovibrio desulfuricans* |
| **935R** | [CP048029.1](https://www.ncbi.nlm.nih.gov/nucleotide/CP048029.1?report=genbank&log$=nuclalign&blast_rank=1&RID=E4D994YU016) | *Chromatiaceae bacterium* |
|  | [CP019225.1, CP028094.1, CP018092.1, CP012832.1, AP012278.1, AP012277.1, AP012276.1, AP012205.1, BA000022.2](https://www.ncbi.nlm.nih.gov/nucleotide/CP019225.1?report=genbank&log$=nuclalign&blast_rank=2&RID=E4D994YU016) | *Synechocystis sp.* |
|  | [CP046913.1, CP046909.1, CP038148.1, CP029640.1](https://www.ncbi.nlm.nih.gov/nucleotide/CP046913.1?report=genbank&log$=nuclalign&blast_rank=3&RID=E4D994YU016) | *Paraburkholderia sp.* |
|  | [CP042829.1](https://www.ncbi.nlm.nih.gov/nucleotide/CP042829.1?report=genbank&log$=nuclalign&blast_rank=5&RID=E4D994YU016) | *Tepidiforma bonchosmolovskayae* |
|  | [CP040098.1](https://www.ncbi.nlm.nih.gov/nucleotide/CP040098.1?report=genbank&log$=nuclalign&blast_rank=6&RID=E4D994YU016) | *Desulfoglaeba alkanexedens* |
|  | [LR134328.1](https://www.ncbi.nlm.nih.gov/nucleotide/LR134328.1?report=genbank&log$=nuclalign&blast_rank=8&RID=E4D994YU016) | *Bacillus freudenreichii* |
|  | [CP029237.1, CP029236.1, CP000910.1](https://www.ncbi.nlm.nih.gov/nucleotide/CP029237.1?report=genbank&log$=nuclalign&blast_rank=9&RID=E4D994YU016) | *Renibacterium salmoninarum* |
|  | [CP019698.1](https://www.ncbi.nlm.nih.gov/nucleotide/CP019698.1?report=genbank&log$=nuclalign&blast_rank=14&RID=E4D994YU016) | *Desulfotomaculum ferrireducens* |
|  | [CP012502.1](https://www.ncbi.nlm.nih.gov/nucleotide/CP012502.1?report=genbank&log$=nuclalign&blast_rank=15&RID=E4D994YU016) | *Bacillus beveridgei* |
|  | [AH013669.2, CP002042.1](https://www.ncbi.nlm.nih.gov/nucleotide/AH013669.2?report=genbank&log$=nuclalign&blast_rank=16&RID=E4D994YU016) | *Meiothermus silvanus* |
|  | [AP017375.1](https://www.ncbi.nlm.nih.gov/nucleotide/AP017375.1?report=genbank&log$=nuclalign&blast_rank=17&RID=E4D994YU016) | *Stanieria sp.* |
|  | [AP012495.1](https://www.ncbi.nlm.nih.gov/nucleotide/AP012495.1?report=genbank&log$=nuclalign&blast_rank=20&RID=E4D994YU016) | *Bacillus subtilis* |
|  | [AP012320.1](https://www.ncbi.nlm.nih.gov/nucleotide/AP012320.1?report=genbank&log$=nuclalign&blast_rank=21&RID=E4D994YU016) | *Rubrivivax gelatinosus* |
|  | [FP929052.1](https://www.ncbi.nlm.nih.gov/nucleotide/FP929052.1?report=genbank&log$=nuclalign&blast_rank=27&RID=E4D994YU016) | *Ruminococcus champanellensis* |
|  | [CP001738.1](https://www.ncbi.nlm.nih.gov/nucleotide/CP001738.1?report=genbank&log$=nuclalign&blast_rank=28&RID=E4D994YU016) | *Thermomonospora curvata* |
|  | [CP001712.1](https://www.ncbi.nlm.nih.gov/nucleotide/CP001712.1?report=genbank&log$=nuclalign&blast_rank=29&RID=E4D994YU016) | *Robiginitalea biformata* |

**Tab. S3.** Name and location of the stations as used for the statistical analyses.

**Tab. S4**. Results from permutation analysis of variance (PERMANOVA) of Bray Curtis dissimilarity between physical and chemical characteristics of the different stations (See figure S2)

Df SumsOfSqs MeanSqs F.Model R2 Pr(>F)

Sampling location 2 3.7471 1.87353 20.4327 0.67198 0.001 ***

Sampling depth 1 0.1786 0.17864 1.9482 0.03204 0.124

Residuals 18 1.6505 0.09169 0.29599

Total 21 5.5762 1.00000
